# P-2119. Development of a Machine-Learning-Based Prediction Model for Candidemia Diagnosis

**DOI:** 10.1093/ofid/ofae631.2275

**Published:** 2025-01-29

**Authors:** Chaeryoung Lee, Da Woon Wang, Sung Kwan Hong, Hyun Wook Han, Jong Hun Kim

**Affiliations:** CHA Bundang Medical Center, CHA University, Seongnam, Kyonggi-do, Republic of Korea; CHA University, Seongnam, Kyonggi-do, Republic of Korea; CHA Bundang Medical Center, Seongnam, Kyonggi-do, Republic of Korea; CHA University, Seongnam, Kyonggi-do, Republic of Korea; CHA University, Seongnam, Kyonggi-do, Republic of Korea

## Abstract

**Background:**

Early recognition and prompt initiation of antifungal therapy for candidemia substantially reduce morbidity and mortality. However, differentiating candidemia from bacteremia remains a clinical challenge and often results in delays in antifungal treatment. This study aimed to develop a machine-learning-based prediction model for candidemia.

**Figure 1.**
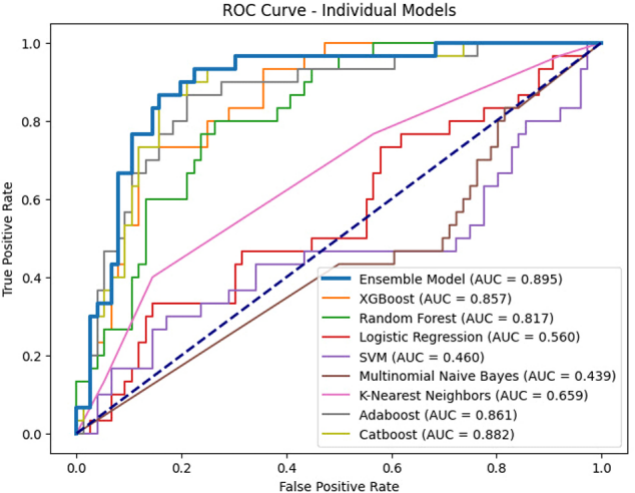
Receiver Operating Characteristic (ROC) Curves for Machine-Learning-Based Prediction Models for Candidemia

**Methods:**

We conducted a single-center retrospective cohort study involving adult patients diagnosed with candidemia from 2014 to 2021, who were included as cases. Control patients, defined as adults with bacteremia, were randomly selected during the same timeframe. Patients with concurrent bacterial and candida infections were excluded. The event time was defined as the moment of blood culture collection for diagnosing each case of candidemia and bacteremia. Clinical data collected at the event time included vital signs (systolic blood pressure and heart rate), epidemiologic, and laboratory data. In total, 55 types of clinical variables were collected. Nine different machine learning algorithms—XGBoost, Random Forest, Logistic Regression, Support Vector Machine, Multinomial Naive Bayes, K-Nearest Neighbors, AdaBoost, CatBoost, and an ensemble incorporating CatBoost and AdaBoost—were utilized to develop a machine-learning-based prediction model for candidemia. The model demonstrating the highest area under the receiver operating characteristic curve (AUROC) was selected for further analysis. Model performance was assessed based on accuracy, precision, recall, and F1 score.

**Results:**

The cohort included 261 patients with candidemia and 522 with bacteremia, with a median age of 68 years. The AUROC for the models varied from 0.439 to 0.895. The ensemble model achieved the highest AUROC of 0.895 (Figure 1). For this ensemble model, the F1 score was 0.853, recall was 0.849, precision was 0.862, and accuracy was 0.849.

**Conclusion:**

A machine-learning-based prediction model could be a useful tool for identifying high-risk patients for the early diagnosis of candidemia.

**Disclosures:**

All Authors: No reported disclosures

